# Investigating the Trajectories of Poor Vision in Children and Adolescents in Wuhan, China From 2016 to 2019: Prospective Cohort Study

**DOI:** 10.2196/53028

**Published:** 2025-02-18

**Authors:** Lijuan Xu, Hanjia Li, Fang Li, Tinghui Zhang, Jingyan Yan, Hong Yan, Lu He, Bin Yu

**Affiliations:** 1 Physical Examination Center, Renmin Hospital of Wuhan University, Wuhan, China; 2 Department of Epidemiology and Health Statistics, School of Public Health, Wuhan University, 115 Donghu Rd, Wuchang District, Wuhan, 430071, China, 86 13797095040; 3 Wuhan Center for Disease Control and Prevention, Wuhan, China; 4 Population and Health Research Center, Wuhan University, Wuhan, China

**Keywords:** children and adolescents, poor vision, group-based trajectory model, myopia, gender difference, China, normal vision group, vision decline group, moderate poor vision group, prevalence

## Abstract

**Background:**

Poor vision is a challenging public health problem among children and adolescents globally and in China. It is well-recognized that early onset of poor vision and progressing to moderate and severe poor vision will increase the risk of irreversible blinding complications. To achieve the national goal of poor vision control and prevention, it is essential to investigate and understand the development of poor vision among children and adolescents in China.

**Objective:**

This study aims to investigate the progression of poor vision among children and adolescents in Wuhan, China, based on a prospective cohort and to provide scientific evidence for the development and implementation of effective poor vision prevention and control programs.

**Methods:**

Data were derived from a 4-year prospective cohort (2016‐2019) of primary and middle school students (N=108,585) in Wuhan, China. Vision condition was measured using the standard logarithmic visual acuity charts. A group-based trajectory model was used to identify trajectories of poor vision overall and by gender and region.

**Results:**

The mean age of the study subjects was 11.13 (SD 3.33) years, 200,110 (53.91%) were male and the majority (354404, 95.48%) were from urban areas. The prevalence of poor vision was 58.51% in 2016, 58.95% in 2017, 53.83% in 2018, and 54.79% in 2019. Group-based trajectory model identified 3 groups, including normal vision group (NVG) (27.4%), vision decline group (VDG) (17.8%), and moderate poor vision group (MPVG) (54.8%). A higher proportion of girls (57.8%) were in the MPV group compared to boys (50.5%), and the VDG showed greater changes in girls compared to boys. Furthermore, urban students (55.3%) had a higher proportion of MPV compared to rural students (47.5%), while urban students (17.2%) had a smaller proportion in the VDG compared to rural students (24%). Further analyses showed that as age increased, the likelihood of being categorized in the NVG decreased (*β*=−.417, *P*<.001), while the likelihood of being in the VDG (*β*=.058, *P*<.001) increased. Compared with boys, girls were more likely to be categorized in the VDG (*β*=.597, *P*<.001) and MPV group (*β*=.362, *P*<.001). Rural students were less likely than urban students to be categorized in the VDG (*β*=−.311, *P*<.001).

**Conclusions:**

The prevalence of poor vision among children and adolescents in Wuhan has remained high over the years, with a slight decrease in recent years. The study identified three groups: normal vision, vision decline, and moderate poor vision. Girls and students from urban areas were more likely to have moderate poor vision, while boys and rural students had a higher proportion of vision decline. These findings provide valuable information for implementing poor vision prevention and control policies in the region.

## Introduction

With the increasing prevalence of poor vision among children and adolescents in recent years, vision problems have been recognized as a major public health challenge globally in the 21st century [1] and in China [2]. Childhood and adolescence are well-accepted as the most vulnerable periods for poor visual development. Visual acuity reaches its full development by approximately 8 years of age, while visual cortex plasticity begins to diminish from the age of 2 years onward [1]. Poor vision can exert significant psychological, educational, and social impacts on children and adolescents, including psychological distress [3], perceived barriers [4], and poor academic achievement [5]. One systematic review and meta-analysis indicated that approximately one-third of children and adolescents were affected by myopia worldwide, and the number was projected to 740 million by 2050 [6] . Another review reported the prevalence of poor vision in children and adolescents as 7.26%, 7.34%, and 2.91% based on uncorrected visual acuity, presenting visual acuity and best corrected visual acuity, respectively, with refractive errors being the most common cause [7].

Compared with other countries, children and adolescents in East Asia reported a higher prevalence of poor vision. For example, data from a literature review indicated that the estimated prevalence of myopia among adolescents in East Asia was 69%, far greater than 16.7% in White adolescents and 14.3% in Hispanic or Latino adolescents [8]. Further, it is well-known that children and adolescents in China experience a significant burden of poor vision. Findings from a systematic review showed that studies from China reported a much higher prevalence of poor vision than those from other countries (13.33%‐41.17% in China vs 1.34%‐29.42% in other countries) [7]. Data from Chinese government indicated that the myopia rate among children and adolescents was 53.6% in 2018,decreased to 50.2% in 2019, and reversed to 52.7% in 2020 [9]. Additionally, data from a review showed that the prevalence of vision impairment in China increased from 19% at age 6 years to 66.9% at age 17 years [10]. To achieve the national goal of controlling and preventing poor vision, it is essential to investigate and understand its development among children and adolescents in China.

It is well-recognized that progression to moderate and severe poor vision increases the risk of irreversible blinding complications, such as retinal detachment, glaucoma, and myopic maculopathy [11]. Previous studies have shown that individuals with early-onset poor vision are at a higher risk of progressing to severe poor vision and vision impairment [12-14]. Therefore, it is valuable to identify the trends of poor vision progression among children and adolescents. However, to the best of our knowledge, most studies among children and adolescents in China have been cross-sectional, limiting the ability to examine the incidence and progression of poor vision [15,16]. Based on a prospective cohort, this study aimed to investigate the progression of poor vision among children and adolescents in China. Findings of the study will provide evidence for the future design and implementation of effective interventions and prevention programs for poor vision.

## Methods

### Participants and Sampling

Study data were derived from a prospective cohort of students in primary and middle schools in Wuchang District, Wuhan City, China. The cohort was developed based on a screening project that included all primary and middle schools in the Wuchang District. All students who were currently studying in the selected schools, with oral or written informed consent collected from the parents or guardians, were recruited for the study. Students who were absent from school on the test day or had severe eye diseases or conditions that may influence the testing results (eg, infections, surgeries) were excluded. Enrollment proportions were 91.10% in 2016, 91.43% in 2017, 95% in 2018, and 96.88% in 2019. The screening team from the Physical Examination Center, Renmin Hospital of Wuhan University visited each primary or middle school on a scheduled day. Schools prepared a classroom for the testing in advance, and students in that school entered the room one class at a time until all students were tested. The screening team completed the screening test for all students in the primary or middle schools in Wuchang District; the process was repeated every year between 2016 and 2019. Wuhan is one of the largest metropolitan cities in central China, with a population of 15 million and a per capita GDP (gross domestic product) of $25,000. Wuchang District is one of the 13 districts of Wuhan and ranks among the largest, with a population of 1.27 million [17]. We aimed to investigate the vision status among students and provide evidence and recommendations for decision-makers. Data from 2016 to 2019 were used in this study, with 89,872, 91,060, 93,391, and 96,849 students enrolled in each year, respectively.

### Ethical Considerations

The original study was a free governmental screening project for which the informed consent was waived from the parents or guardians. This study involved secondary data analysis of screening data and was approved by the Medical Ethics Committee at Wuhan University School of Medicine (WHU-LFMD-IRB2023031). Informed consent for the secondary data analysis was waived, and all data were was deidentified. No compensation was provided to the participants.

### Measurements

#### Vision Status Assessment

Participants’ visual acuity was measured by the standard logarithmic visual acuity charts, which has been widely used in poor vision screening in ophthalmology clinics and schools for over 20 years in China [18]. Participants were asked to stand at a distance of 5 m from the visual acuity chart and identify the direction of the “E optotype.” Visual acuity was recorded using the 5-point method; this is an original visual acuity recording method in China, which stipulates normal vision as ≥5.0 and no light perception as 0. The participant’s visual acuity is the recorded value of visual acuity corresponding to the smallest row for which they can correctly identify more than half the directions of the “E” optotype [18]. All inspectors received professional training.

The screening results were categorized based on the requirements of the technical standard for physical examination for students [19]; normal vision was defined as 5.0 in both left and right eyes. In cases where the visual acuity of left and right eyes differed, the lower value was used for classification. Mild poor vision was defined as a visual acuity of 4.9, moderate poor vision as <4.9 but >4.5, and severe poor vision as ≤4.5.

#### Demographic Variables

Demographic variables, including age (in years), sex (male or female), and place of origin (urban or rural), were provided by the schools where the children and adolescents were enrolled.

### Statistical Analysis

Descriptive analyses (eg, frequency, proportion, mean, standard deviation) were used to summarize the sample characteristics. The group-based trajectory model (GBTM) was used to analyze the longitudinal data, explore the heterogeneity within the study sample, and further identify different developmental trajectories for poor vision. The underlying assumption of the GBTM is that the overall study sample consists of several latent subgroups with distinct developmental trajectories or patterns. Findings from the GBTM could help researchers and decision-makers to have a deeper understanding of the variability within the population and how different individuals progress over time. The GBTM has been widely used in various areas, including behaviors, mental health [20], and medical conditions [21]. It is an application of finite mixture modeling, which can approximate different developmental trajectories with a finite number of groups through maximum likelihood estimation [22]. The model accommodates missing data for certain time points, as demonstrated in previous studies [23,24]. Here, *Y_i_*={*y_i1_,y_i2_,...,y_it_*} denotes individual *i*’s longitudinal sequence of the *k^th^* measured over T=4 time periods, *π_g_* denotes the probability that individual *i* belongs to *g^th^* group, *P^g^(Y_i_*) represents the conditional probability of *Y_i_* for a given membership in group *g*. The probability of the individual *i*’s longitudinal sequence is expressed by Equation 1.


(1) $$P(Y_{i})=\sum_{g=1}^{G}\pi_{g}P^{g}\left(Y_{i}\right)$$

Furthermore, GBTM assumes that the random variables *y_it_*(*t*=1,2,...,T) are independent of each other when within groups. Therefore, we can use Equation 2 to definite *P^g^(Y_i_*).


(2) $$P^{g}\left(Y_{i}\right)=\prod_{t=1}^{T}P^{g}\left(y_{it}\right)$$

We tested models with varying numbers of trajectory groups and orders of polynomial function, and then selected the best model based on four criteria [25-30] : (a) a lower absolute value of Bayesian Information Criteria , (b) the average posterior probability (Avepp) for class membership >0.7 for all groups, (c) all groups included at least 5% of the total sample, and (d) a simplified and rational model. Following the establishment of the foundational model, the incorporation of age as a time-dependent covariate, along with initial age, sex, and place of origin as independent covariates, was undertaken to ascertain the impact of these variables on the progression of visual acuity. All analyses were implemented using SAS (version 9.4; SAS Institute) and R software (version 4.2.1; R Foundation for Statistical Computing).

## Results

### Characteristics of the Study Sample

Results in Table 1 show that among the 89,872 children and adolescents in Wave 1 (2016), 48,431 (53.89%) were male, 83,102 (92.47%) were from urban areas, and the mean age of the sample was 11.24 (SD 3.33) years. Among the 91,060 participants in Wave 2 (2017), 49,056 (53.87%) were male, 85,826 (94.25%) were from urban areas, and the mean age of the sample was 11.16 (SD 3.32) years. Among the 93,391 participants in Wave 3 (2018), 50,350 (53.91%) were male, 90,503 (96.91%) were from urban areas, and the mean age of the sample was 11.09 (SD 3.33) years. In Wave 4 (2019), 52,273 (53.97%) were male, 94,973 (98.06%) were from urban areas, and the mean age of the sample was 11.04 (SD 3.33) years.

**Table 1.** Characteristics of the children and adolescents in Wuhan, China, between Wave 1 in 2016‐Wave 4 in 2019.

Variables	Number of participants between Wave I (2016) and Wave IV (2019)			
	Wave 1 (n=89,872)	Wave 2 (n=91,060)	Wave 3 (n=93,391)	Wave 4 (96,849)
Sex, n (%)				
Male	48,431 (53.89)	49,056 (53.87)	50,350 (53.91)	52,273 (53.97)
Female	41,441 (46.11)	42,004 (46.13)	43,041 (46.09)	44,576 (46.03)
Age (years), mean (SD)	11.24 (3.33)	11.16 (3.32)	11.09 (3.33)	11.04 (3.33)
Grade, n (%)				
Grade 1	9161 (10.19)	9350 (10.27)	10,460 (11.2)	10,659 (11.01)
Grade 2	8878 (9.88)	9224 (10.13)	9253 (9.91)	10,649 (11)
Grade 3	8663 (9.64)	8966 (9.85)	9140 (9.79)	9170 (9.47)
Grade 4	7788 (8.67)	8609 (9.45)	8844 (9.47)	9190 (9.49)
Grade 5	7953 (8.85)	7672 (8.43)	8667 (9.28)	8878 (9.17)
Grade 6	7690 (8.56)	7972 (8.75)	7774 (8.32)	8698 (8.98)
Grade 7	7777 (8.65)	7757 (8.52)	7762 (8.31)	7735 (7.99)
Grade 8	7665 (8.53)	7713 (8.47)	7640 (8.18)	7715 (7.97)
Grade 9	7671 (8.54)	7513 (8.25)	7552 (8.09)	7479 (7.72)
Grade 10	5795 (6.45)	5722 (6.28)	5692 (6.1)	6005 (6.2)
Grade 11	5715 (6.36)	5718 (6.28)	5625 (6.02)	5701 (5.89)
Grade 12	5116 (5.69)	4844 (5.32)	4979 (5.33)	4944 (5.11)
Region, n (%)				
Rural	6770 (7.53)	5234 (5.75)	2888 (3.09)	1876 (1.94)
Urban	83,102 (92.47)	85,826 (94.25)	90,503 (96.91)	94,973 (98.06)

### Prevalence of Poor Vision

Results in Table 2 show that the prevalence of poor vision in the study sample was 58.51% in 2016, 58.95% in 2017, 53.83% in 2018, and 54.79% in 2019. The prevalence of poor vision from 2016 to 2019 ranged from 12.71% to 12.32% for mild poor vision, from 20.46% to 21.05% for moderate poor vision, and from 25.34% to 21.42% for severe poor vision. Additional information by gender and region is presented in Table 2.

**Table 2.** Prevalence of poor vision among children and adolescents in Wuhan, China, from 2016 to 2019.

Vision condition	Number of participants between 2016 and 2019			
	2016 (N=82,477)	2017 (N=90,396)	2018 (N=91,548)	2019 (N=93,068)
Overall, n (%)				
Normal	34,224 (41.49)	37,107 (41.05)	42,266 (46.17)	42,079 (45.21)
Mild poor vision	10,480 (12.71)	10,389 (11.49)	9211 (10.06)	11,467 (12.32)
Moderate poor vision	16,873 (20.46)	18,145 (20.07)	17,622 (19.25)	19,589 (21.05)
Severe poor vision	20,900 (25.34)	24,755 (27.39)	22,449 (24.52)	19,933 (21.42)
Males, n (%)				
Normal	19,737 (44.13)	21,303 (43.77)	24,142 (48.88)	23,969 (47.67)
Mild poor vision	5422 (12.12)	5382 (11.06)	4838 (9.8)	5928 (11.79)
Moderate poor vision	9075 (20.29)	9596 (19.72)	9320 (78.87)	10,402 (20.69)
Severe poor vision	10,491 (23.46)	12,387 (15.45)	11,087 (22.45)	9980 (19.85)
Females, n (%)				
Normal	14,487 (38.37)	15,804 (37.87)	18,124 (42.99)	18,110 (42.32)
Mild poor vision	5058 (13.4)	5007 (12)	4373 (10.37)	5539 (12.94)
Moderate poor vision	7798 (20.66)	8549 (20.49)	8302 (19.69)	9187 (21.47)
Severe poor vision	10,409 (27.57)	12,368 (29.64)	11362 (26.95)	9953 (23.26)
Urban students, n (%)				
Normal	30,985 (40.76)	34,530 (40.54)	40,703 (45.91)	41,251 (45.19)
Mild poor vision	9616 (12.65)	9894 (11.62)	8932 (10.07)	11,252 (12.33)
Moderate poor vision	15,567 (20.48)	17,081 (20.06)	16,997 (19.17)	19,179 (21.01)
Severe poor vision	19,852 (26.11)	23,665 (27.79)	22,034 (24.85)	19,596 (21.47)
Urban males, n (%)				
Normal	17,757 (43.37)	19,727 (43.23)	23,159 (48.54)	23,450 (47.62)
Mild poor	4940 (12.07)	5106 (11.19)	4681 (9.81)	5817 (11.81)
Moderate poor vision	8307 (20.29)	8991 (19.7)	8997 (18.86)	10,162 (20.64)
Severe poor vision	9938 (24.27)	11,811 (25.88)	10,879 (22.8)	9816 (19.93)
Urban females, n (%)				
Normal	13,228 (37.71)	14,803 (37.44)	17,544 (42.84)	17,801 (42.35)
Mild poor vision	4676 (13.33)	4788 (12.11)	4251 (10.38)	5435 (12.93)
Moderate poor vision	7260 (20.7)	8090 (20.46)	8000 (19.54)	9017 (21.45)
Severe poor vision	9914 (28.26)	11854 (29.98)	11,155 (27.24)	9780 (23.27)
Rural students, n (%)				
Normal	3239 (50.16)	2577 (49.31)	1563 (54.23)	828 (46.26)
Mild poor vision	864 (13.38)	495 (9.47)	279 (9.68)	215 (12.01)
Moderate poor vision	1306 (20.23)	1064 (20.36)	625 (21.69)	410 (22.91)
Severe poor vision	1048 (16.23)	1090 (20.86)	415 (14.4)	337 (18.83)
Rural males, n (%)				
Normal	1980 (52.34)	1576 (51.96)	983 (58.82)	519 (50.19)
Mild poor vision	482 (12.74)	276 (9.1)	157 (9.4)	111 (10.74)
Moderate poor vision	768 (20.3)	605 (19.95)	323 (19.33)	240 (23.21)
Severe poor vision	553 (14.62)	576 (18.99)	208 (12.45)	164 (15.86)
Rural, females, n (%)				
Normal	1259 (47.08)	1001 (45.64)	580 (47.89)	309 (40.87)
Mild poor vision	382 (14.29)	219 (9.99)	122 (10.07)	104 (13.76)
Moderate poor vision	538 (20.12)	459 (20.93)	302 (24.94)	170 (22.49)
Severe poor vision	495 (18.51)	514 (23.44)	207 (17.09)	173 (22.88)

### Trajectories of Poor Vision

**Figure 1.**
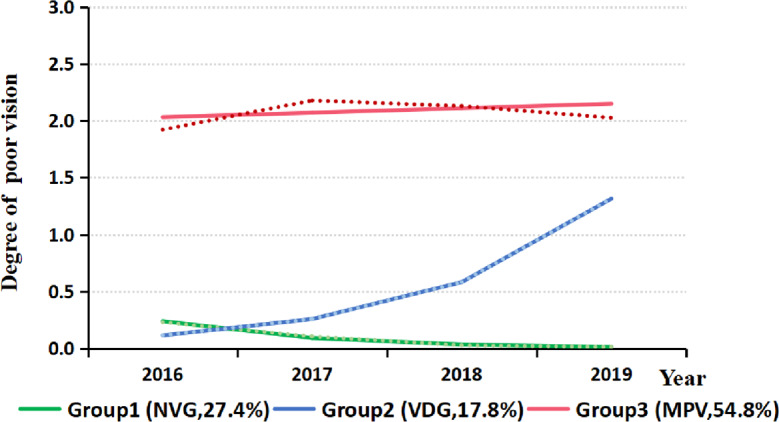
Trajectories of poor vision among children and adolescents in Wuhan, China, 2016‐2019. Dotted lines represent the actual value, solid lines represent the predicted value. NVG: normal vision group, VDG: vision decline group, MPV: moderate poor vision group.

Figure 1 shows the three distinct trajectories that were identified based on the lowest absolute Bayesian Information Criteria value (−361460.5). Group 1 (n=29752, 27.4%; Avepp=0.76) was characterized by a low degree of poor vision with a reduction in first 2 years and was named the normal vision group (NVG). Group 2 (n=19328, 17.8%; Avepp=0.72) was characterized by a rapid increase in poor vision and was named the vision decline group (VDG). Group 3 (n=59505, 54.8%; Avepp=0.93) was characterized by a stable level of moderate poor vision and was named the moderate poor vision group (MPV).

**Figure 2.**
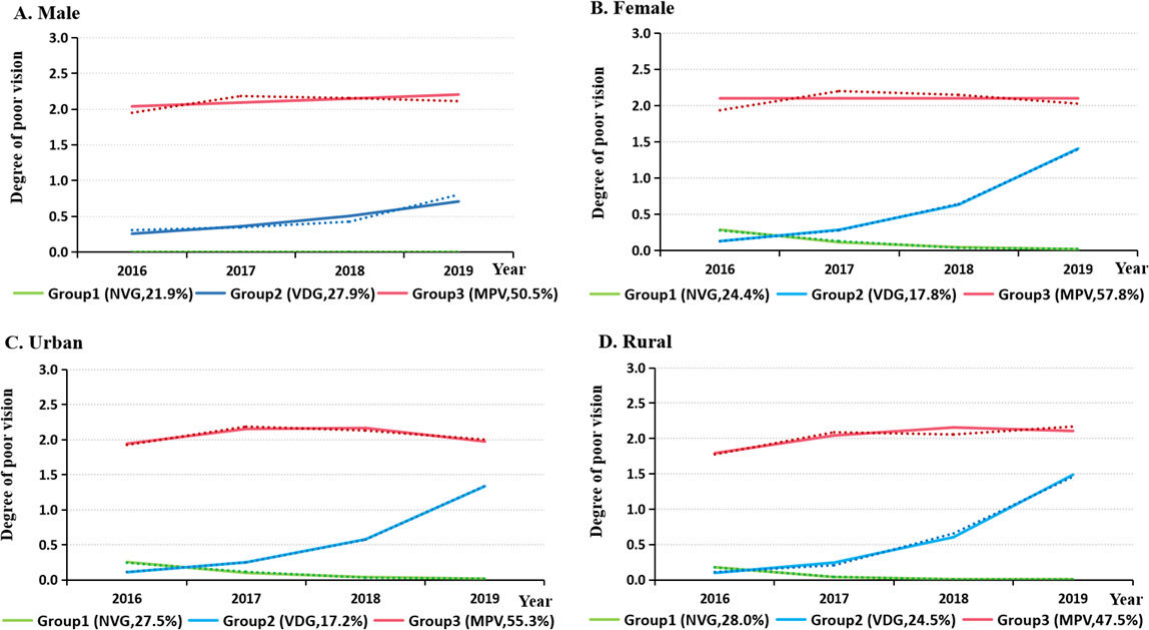
Trajectories of poor vision among children and adolescents in Wuhan, China, by sex and region between 2016-2019. Dotted lines represent the actual value, solid lines represent the predicted value. NVG: normal vision group; VDG: vision decline group; MPV: moderate poor vision group.

Figure 2 shows the trajectories of poor vision by sex and region, with a total of three trajectories identified. Compared to males, females had a higher proportion in Group 3 (57.8% vs 50.5%), and a lower proportion in Group 2 (17.8% vs 27.9%). Compared to rural areas, students in the urban areas had a higher proportion in Group 3 (55.3% vs 47.5%) and a lower proportion in Group 2 (17.2% vs 24.5%).

**Figure 3.**
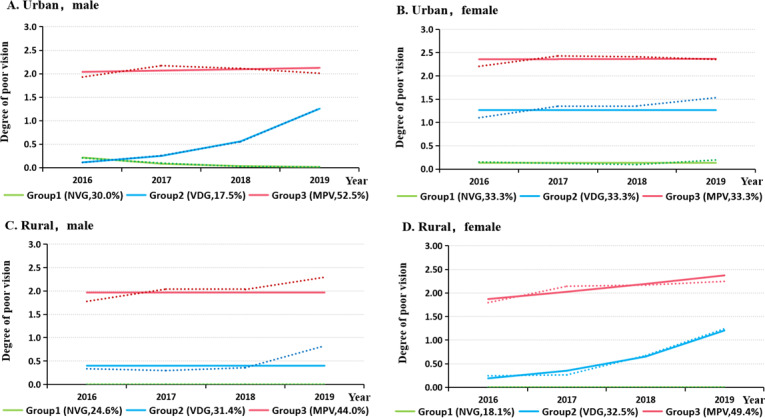
Trajectories of poor vision among children and adolescents in Wuhan, China, 2016‐2019, across sex and regions. Dotted lines represent the actual value, solid lines represent the predicted value. NVG: normal vision group, VDG: vision decline group, MPV: moderate poor vision group.

Figure 3 shows the trajectories of poor vision by the interaction of sex and region. The highest proportion in Group 3 was found among urban females (56.8%), while the lowest proportion was found in rural males (43.6%). The highest proportion in Group 2 was found among rural males (32.8%), followed by urban females (27.2%), rural females (25.8%), and urban males (17.5%).

### Factors Influencing Trajectory Membership and Progression

Data in Table 3 show that with increasing age, students were less likely to be categorized into Group 1 (*β*=−0.417, *P*<.001), and more likely to be categorized into Group 2 (*β*=.058,<.001). For group membership (Group 1 was used as reference), compared to boys, girls were more likely to be categorized into Group 2 (*β*=.597, *P*<.001) and Group 3 (*β*=.362, *P*<.001). Additionally, compared to urban students, students from rural area were less likely to be categorized into Group 2 (*β*=−.311, *P*<.001). Further, with increasing initial enrollment age, students were more likely to be categorized into Group 1 (*β*=.369, *P*<.001) and Group 3 (*β*=.096, *P*<.001).

**Table 3.** Factors influencing trajectory membership and progression of poor vision among children and adolescents in Wuhan, China between 2016 and 2019.

Group	Variables	β	*t* test (*df*)^a^	*P* value
Trajectories progression				
Group 1^b^	Age	−.417	23.730 (85,820)	<.001
Group 2^c^	Age	.058	58.250 (85,820)	<.001
Group 3^d^	Age	.002	0.208 (85,820)	.84
Group membership (ref: Group 1)				
Group 2	Sex, (ref:Boys)			
	Girls	.597	22.709 (49,894)	<.001
	Region (ref:Urban)			
	Rural	−.311	−6.973 (5,092)	<.001
	Initial age	.369	33.711 (84,929)	<.001
Group 3	Sex (ref:Boys)			
	Females	.362	9.338 (49,994)	<.001
	Region (ref:Urban)			
	Rural	−.007	−0.113 (5,092)	.910
	Initial age	.096	5.391 (84,929)	<.001

a 2-tailed t test was applied in the analysis.

b Group 1: normal vision group (NVG).

c Group 2: vision decline group (VDG).

d Group 3: moderate poor vision group (MPV).

## Discussion

### Principal Findings

This study investigated the progression of poor vision by analyzing data from a prospective cohort of primary and middle school students in Wuhan, China. To the best of our knowledge, this is the first study to use longitudinal data to identify the progression pattern of poor vision among children and adolescents. The findings of the study deepen our understanding of the development of poor vision and provide valuable evidence for devising and implementing intervention programs.

Population-based research indicates that poor vision in most school children results from uncorrected refractive errors [31,32]. Visual acuity has been widely used as a proxy measure for refractive error in children in previous studies [33-35] and is useful for estimating the prevalence of myopia in large populations that undergo vision screening when refraction is not feasible [36]. In this study, we found that the prevalence of poor vision among children and adolescents was 58.51% in 2016, 58.95% in 2017, 53.83% in 2018, and 54.79% in 2019, showing a decline in the latter two years. It is well-recognized that Chinese students are under greater academic pressure, spend more time on near-work activities and less time on outdoor activities, which may contribute to the high rates of poor vision [28]. The declines observed in the latter two years may be associated with the nationwide policy change regarding myopia prevention and control [29].

In this study, we identified 3 different development trajectories of poor vision among children and adolescents. Notably, more than half of the participants were categorized into the progression pattern of MPV during 2016 and 2019, amounting to 49,880 primary and middle school students in Wuchang district. If this pattern was applied to Wuhan City, approximately 589,135 primary and middle students would be fall into the MPV category. Meanwhile, our results show that 17.8% of the students enrolled were categorized into the VDG, which was characterized by a progression from normal vision to MPV. This finding suggests that the magnitude of vision loss is increasing every year. A study conducted by Ma et al [10] also showed that the prevalence rate of vision impairment was associated with grade and age in a nonlinear pattern. The underlying mechanisms of the rapid increasing pattern from normal to moderate poor vision may include high academic stress, long time of study, frequent use of electronic devices, lack of outdoor physical activities, among others [28,37-40]. These findings also highlight the urgent need for effective intervention and prevention programs to control poor vision development; potential actions include encouraging students to play outdoor sports, reducing their academic stress, and limiting time spent on electronic devices [41,42]. Further, we found that 27.4% of the participants maintained relatively normal vision during the study period. Future studies should investigate the characteristics of this group and explore the potential protective factors against poor vision.

This study also suggests that being girls are at a higher risk of poor vision than boys, with the average poor vision rate being 59.61% in girls compared to 53.89% in boys, consistent with previous studies [26,43]. Moreover, a study on nationally representative samples conducted by Li and Mo [39] reported that 64.49% of girls were myopic while 54.44% of the boys. This trend was further confirmed in the trajectory analysis, where a greater proportion of females were classified into the MPV group compared to males. Although fewer girls belonged to the VDG, female children and adolescents experienced a greater decline in vision than males. The findings of this study indicated that girls had a greater risk of being assigned to both the VDG and the MPV than to the NVG; this result may be attributed to the fact that girls may spend less time on outdoor activities and more time on near-work tasks than boys [28,44,45].

Further, study findings suggest that urban students had a higher risk of poor vision than youth from rural areas, with a higher prevalence of poor vision and a greater proportion in the MPV category. Compared to urban students, rural children, and adolescents were less likely to be classified into the VDG than the NVG. Previous studies have similarly demonstrated higher rates of poor vision among urban students than those in rural areas [46]. This phenomenon could result from higher academic pressure, more time spent using electronic devices, and less outdoor activities among urban students [47,48].

### Limitations

Although this study included a large population, it had several limitations. First, the results of the standard for logarithmic visual acuity charts were used as the sole criterion to determine the visual acuity of participants, and the cycloplegic refraction was not measured, which may have introduced measurement errors. We used the term poor vision instead of myopia throughout the paper to avoid the overestimation. Second, this study was only performed in a single district in Wuhan, and caution should be exercised when generalizing the findings to other areas. Third, there is a lack of data on demographic variables and risk factors for poor vision, that limited further analysis.

### Conclusions

This study identified 3 groups with distinct poor vision progression patterns, including normal vision, visual decline, and MPV. Furthermore, the prevalence of poor vision was higher in girls than boys and higher in urban than rural students. Therefore, particular attention should be paid to girls and urban students for the prevention and control of poor vision among primary and secondary school students in the future.

## Abbreviations

Avepp: average posterior probability
GBTM: Group-based trajectory model
GDP: gross domestic product
MPV: moderate poor vision
NVG: normal vision group
VDG: vision decline group
